# Emergency Visits and Hospitalization After Chat Message, Voice Call, or Video Call for Telehealth in Obstetrics and Gynecology Using Telehealth Service User Data in Japan: Cross-sectional Study

**DOI:** 10.2196/35643

**Published:** 2022-09-23

**Authors:** Koichi Sakakibara, Daisuke Shigemi, Rena Toriumi, Ai Ota, Nobuaki Michihata, Hideo Yasunaga

**Affiliations:** 1 Department of Health, Behavior, and Society Johns Hopkins Bloomberg School of Public Health Baltimore, MD United States; 2 Kids Public Inc Tokyo Japan; 3 Department of Health Services Research Graduate School of Medicine The University of Tokyo Tokyo Japan; 4 Department of Clinical Epidemiology and Health Economics School of Public Health The University of Tokyo Tokyo Japan

**Keywords:** eHealth, gynecology, chat message, mobile health, mHealth, obstetrics, safety, telehealth, telemedicine, video call, voice call

## Abstract

**Background:**

In obstetric and gynecologic practices, synchronous telehealth services via chat message, voice calls, and video calls have been increasingly equipped to improve patients’ health care accessibility and clinical outcomes. Nevertheless, differences in clinical outcomes between communication tools remain unknown, especially in terms of safety.

**Objective:**

This study compared the occurrence of emergency visits and hospitalization after telehealth services through different communication tools, including chat messages, voice calls, and video calls.

**Methods:**

We collected data on obstetric and gynecologic concerns of women who consulted specialized doctors and midwives through a telehealth consulting service in Japan (Sanfujin-ka Online) between January 1, 2019, and December 31, 2020. The outcomes were emergency visits or hospitalizations at night after the consultation. Chi-square test and multivariate logistic regression analysis were performed to compare the clinical outcomes between the groups who received telehealth services via chat message, voice calls, and video calls.

**Results:**

This study included 3635 participants. The mean age of the participants was 31.4 (SD 5.7) years, and the largest age group (n=2154, 59.3%) was 30-39 years. The numbers (or proportions) of those who received telehealth services via chat message, voice calls, and video calls were 1584 (43.5%), 1947 (53.6%), and 104 (2.9%), respectively. The overall incidence of the outcome was 0.7% (26/3635), including 10 (0.3%) cases of chat message, 16 (0.5%) cases of voice calls, and no video calls. There were no emergency visits that happened due to inappropriate advice. No significant difference in the proportions of the outcomes was observed between the communication tools (*P*=.55). The multivariate logistic regression analysis showed no significant differences in the outcome between those who used chat message and those who used voice calls (odds ratio 1.63, 95% CI 0.73-3.65).

**Conclusions:**

The communication tools of telehealth services in obstetrics and gynecology did not show a significant difference in terms of emergency visits or hospitalizations after using the service.

## Introduction

Telehealth refers to various health care services that use digital information and communication technologies. They are categorized into synchronous or asynchronous approaches; synchronous approaches comprise real-time virtual communication and monitoring by health care professionals, whereas asynchronous approaches, also called store-and-forward telehealth, use electronic recording and screening of patient-generated health data [1]. The World Health Organization has highlighted telehealth’s potential to improve patients’ access to health care services and reduce medical costs [2]. The demand for telehealth services has been expanding, especially in response to the COVID-19 pandemic and physical movement restrictions [3].

In obstetric and gynecologic practices, telehealth has been increasingly equipped to improve patients’ accessibility to health care services. Past studies have demonstrated the effectiveness of telehealth services in obstetrics and gynecology, including an improvement in health behaviors [4,5], weight management [6], mental health issues [7], and postpartum depression [8] in pregnant women. Another review has reported the association between telehealth and the improvement of health outcomes in high-risk obstetric patients, such as women with hypertensive disorders, diabetes mellitus, fetal anomalies, and pregnancy in underserved areas [9].

Despite the accumulated knowledge of telehealth in obstetrics and gynecology, scant attention has been paid to safety per the service characteristics. It is especially critical to closely evaluate the safety of synchronous telehealth services, as medical practitioners directly intervene in patients’ medical behaviors, while communication via synchronous telehealth tools may limit the information that a medical practitioner can obtain from a patient. The existing literature has reported no significant difference in health outcomes between synchronous telehealth services and traditional in-person services. For example, the Antenatal and Neonatal Guidelines, Education, and Learning System, one of the largest telehealth obstetric care programs providing a phone consultation service for pregnant women in the United States, was as effective as traditional in-person services [10,11]. Text-4-Baby is another telehealth service for pregnant women, and its text messaging service was associated with an improvement in women’s health behaviors and attitudes toward child-rearing [12,13]. Additionally, programs with synchronous videoconferences were not associated with increased risks of health complications and medical adherence in comparison with traditional outreach programs [14,15].

The remaining knowledge gap is the comparison between communication tools. Each communication tool (ie, chat message, voice calls, and video calls) may provide different amounts and quality of patients’ information to medical practitioners, which can result in divergence in interventions and health outcomes. Thus, we analyzed the secondary data of an online synchronous medical consultation service to compare and evaluate the safety of communication tools (ie, chat message, voice calls, and video calls). Although there are no established safety indicators for online consultations, we defined emergency visits and hospitalization as postconsultation clinical outcomes because emergency hospital visits resulting from inappropriate advice at the time of telehealth services are an important clinical concern.

This study analyzed data from Kids Public Inc, a Japanese health care company providing an online health consultation service in obstetrics and gynecology (Sanfujin-ka Online). The service allows women to consult specialized doctors and midwives about obstetric and gynecologic concerns. Women can use this consultation service at any time before and during pregnancy as well as after childbirth. In Japan, there are no restrictions or regulations regarding the provision or use of telehealth consultations based on the weeks of pregnancy. The users complete a medical questionnaire before each consultation, and medical professionals monitor the urgency based on their responses and the consultations. This service provides only consultations with medical professionals and does not offer medical services such as diagnosis or prescription. Therefore, apart from questions regarding symptoms and medical examinations, the service receives consultations regarding small concerns and questions about health-related daily life issues. In total, 171 medical professionals (ie, obstetrician-gynecologists and midwives) were registered as consultants and responded to consultation requests from 6 PM to 10 PM on weekdays. As Kids Public Inc mainly works with corporations and local governments, most users can avail of the service without payment. This service was approved as a commissioned project by the Ministry of Economy, Trade and Industry in Japan in May 2020 and provided free service to all Japanese citizens until the end of August 2020.

## Methods

### Recruitment and Data Description

We collected data from Kids Public Inc users who responded to a survey between January 1, 2019, and December 31, 2020. Kids Public Inc developed the web-based survey, and some authors were involved in its development as members of Kids Public Inc. This voluntary survey was sent to the users via email automatically within 24 hours of the consultation. The survey was password protected, and service users could carry out data entry via the internet. Other than this one-time self-reporting survey, no protocols for monitoring the patients’ postconsultation health behaviors and hospital visits were implemented. There were no incentives for respondents, but they were allowed to skip the questions if they were not comfortable with answering. The data collected through the survey contained the communication tools for consultation (ie, chat message, voice call, or video call), emergency visits or hospitalization at night after the consultation, the consultant category (ie, obstetrician or midwife), the user’s situation at the time of consultation (eg, pregnancy, post partum, or other), and whether the hospital visit was unexpected. Data on users’ age were also collected because several studies on telehealth services during the pandemic identified that older age was associated with lower use of digital health services [16,17]. Although the demographic distribution of telehealth service use has not been closely explored in Japan, the government report in 2021 addressed that age disparities may have been critical in telehealth use [18]. Consent to use the data anonymously was obtained from all participants at the time of consultation.

### Statistical Analysis

We conducted a secondary analysis of anonymized data to examine differences in the proportion of emergency visits or hospitalizations after consultations according to the communication tools (ie, chat message, voice call, or video call). The exposures, in this study, were the 3 types of communication tools, and the outcomes were the emergency visits or hospitalizations at night after the consultation. Further, consultation records were reviewed to assess whether the emergency visits were unexpected and whether they were caused by inappropriate advice. A chi-square test was used to compare the proportions of the groups. Additionally, multivariate logistic regression analysis was performed with the outcome as the objective variable. Other acquired information was used as a covariate to adjust the users’ background. Odds ratios (ORs) and 95% CIs were calculated. The model’s goodness of fit was confirmed using the Hosmer-Lemeshow test. All values included in the multivariate analysis were evaluated for correlations, and the absence of multicollinearity was confirmed.

All statistical analyses were performed using Stata software (version 16.0; StataCorp LP). All 95% CIs and *P* values were based on 2-sided hypothesis tests, where *P*<.05 was considered to denote statistical significance.

### Ethics Approval

This study was approved by the Institutional Review Board of the University of Tokyo for joint research between Kids Public Inc and the University of Tokyo (number 2020043NI). Two faculty members of the Department of Clinical Epidemiology and Health Economics at the Graduate School of Medicine, University of Tokyo, conducted and confirmed the analyses to ensure the study’s neutrality and transparency. Moreover, CHERRIES checklist was followed, as this is a useful guideline for investigators reporting results of web surveys [19].

## Results

We collected 3635 responses from the web-based survey after the consultations, and the response rate was 40.9%. Table 1 shows the characteristics of the eligible participants. As for the communication tools, voice call was the most common (n=1947, 53.6%), followed by chat message (n=1584, 43.5%). Video call was used by a small proportion of participants (n=104, 2.9%). Overall, the mean age was 31.4 (SD 5.7) years, and the largest age group (n=2154, 59.3%) was 30-39 years. Women under 30 years of age tended to choose chat message. During the consultation, more than half of the participants (n=1883, 51.8%) were in the postpartum period, followed by participants during pregnancy (n=1062, 29.2%), and others (n=690, 19%). Women in postpartum period were the most common users of each tool, but pregnant women were relatively more common in the chat message group. Approximately 60% (n=2174) of the participants consulted obstetrician-gynecologists. In the chat message group, most consultations were with doctors, whereas, in the video call group, most consultations were with midwives. In the voice call group, the proportion of doctors and midwives was almost equal.

Table 2 shows the incidence of primary outcomes divided by communication tools. Of the 3635 responses, 26 (0.7%) cases were reported, including 16 (0.5%) emergency hospital visits and 10 (0.3%) emergency hospitalizations. Of these 26 cases, 10 (0.7%) used chat message, and 16 (0.8%) used voice calls. No outcome incidence was observed among participants using video calls. No significant differences in outcome incidence between communication tools were observed (*P*=.55).

**Table 1:** Participants’ characteristics (N=3635).

Variables		Total (N=3635), n (%)	Chat message (n=1584, 43.5), n (%)	Voice call (n=1947, 53.6), n (%)	Video call (n=104, 2.9), n (%)	*P* value	
**Age (years)**							<.001
	<20	130 (3.6)	73 (4.6)	54 (2.8)	3 (2.9)		
	20-29	1103 (30.3)	517 (32.6)	564 (29)	21 (20.2)		
	30-39	2154 (59.3)	905 (57.1)	1178 (60.5)	72 (69.2)		
	≥40	248 (6.8)	89 (5.6)	151 (7.8)	8 (7.7)		
**Perinatal situation**							<.001
	Pregnant	1062 (29.2)	533 (33.7)	518 (26.6)	11 (10.6)		
	Post partum	1883 (51.8)	712 (44.9)	1088 (55.9)	83 (79.8)		
	Other	690 (19)	339 (21.4)	341 (17.5)	10 (9.6)		
**Consultant**							<.001
	Doctor (obstetrician-gynecologist)	2174 (59.8)	1129 (71.3)	1006 (51.7)	39 (37.5)		
	Midwife	1461 (40.2)	455 (28.7)	941 (48.3)	65 (62.5)		

**Table 2:** Incidence of emergency night visits or hospitalizations within 24 hours after the consultation via communication tools (N=26).

Variables		Participants, n (%)	*P* value
**Communication tool**			.55
	Chat message	10 (0.7)	
	Voice call	16 (0.8)	
	Video call	0 (0)	

Table 3 shows the results of the multivariate logistic regression analysis of the primary outcomes. No significant difference in the OR for outcome incidence was observed between chat message and voice calls. Video calls were not included in the regression analysis because the number of outcome occurrences was zero. No significant association was shown with age (OR 0.97, 95% CI 0.90-1.05), consultant occupation (OR 0.63, 95% CI 0.17-2.31), or postpartum period (OR 0.57, 95% CI 0.11-2.96) at the time of consultation, while pregnancy status was significantly associated with the outcome (OR 3.54, 95% CI 1.11-11.3).

In all cases that resulted in emergency visits or hospitalizations, the consulted physician or midwife explained the necessity of emergency visits or seeing a doctor when symptoms worsened, which meant there were no emergency visits or hospitalizations.

**Table 3:** The multivariate logistic regression analysis of emergency night visits or hospitalizations within 24 hours after consultation via communication tools.

Variables			OR^a^ (95% CI)
Age^b^ (years)			0.968 (0.869-1.05)
**Communication tool**			
	Chat message	Ref^c^	
	Voice call	1.63 (0.731-3.65)	
**Perinatal situation**			
	Pregnant	3.54 (1.11-11.3)	
	Post partum	0.57 (0.11-2.96)	
	Other	Ref (1.08-2.03)	
**Consultant**			
	Doctor (obstetrician-gynecologist)	Ref	
	Midwife	0.63 (0.17-2.31)	

^a^OR: odds ratio.

^b^Continuous variable.

^c^Ref: reference group.

## Discussion

### Principal Results

This study was designed to investigate the safety of online consulting services in obstetrics and gynecology using communication tools (ie, chat message, voice calls, and video calls). Of 3635 collected samples from an online consultation service, 26 (0.7%) cases of emergency visits or hospitalization at night after consultation use were reported. There was no significant difference in the primary outcome incidence between communication tools. Additionally, the results of the multivariate logistic regression analysis indicated no significant differences in the OR of outcome incidence between chat message and voice calls, even when adjusting for the participants’ age, consultants’ occupation, and participants’ situation at the time of consultation. The findings of this study suggest that there are no significant differences in clinical safety among communication tools. However, video calls could not be evaluated in the regression analysis because the number of outcome occurrences was zero. Moreover, the low rate of hospital visits after service use could be attributed to the characteristics of the service; this telehealth service provides only consultations with medical professionals and does not offer medical services such as diagnosis or prescription, so the main users tend to be women who have small concerns and daily-life issues rather than those who have visible symptoms that potentially require hospital visits. Although the service monitors the level of urgency through the completion of a questionnaire before service use and online consultation with a specialist, this finding suggest that the monitoring protocols and method of evaluating consultations after service use may need to be improved. It is important to design service improvements and further research based on this finding.

In this study, users were allowed to choose one of three communication tools, and most participants selected either voice calls (n=1947, 53.6%) or chat message (n=1584, 43.5%). The proportion of video calls was very low (n=104, 2.9%). These results indicate the importance of setting up remote consultation services equipped with a variety of communication tools. In the field of obstetrics and gynecology, video calls were not favored by users, suggesting low compatibility with online consultations. In addition, differences in user background were observed among the three tools: young people under 30 years and pregnant women chose chat message more often. From this perspective, it is preferable to have a variety of communication tools.

The novelty of our study is that we have shown the clinical safety of online obstetric and gynecologic consulting services. Despite the emerging knowledge about synchronous telehealth services, few studies have addressed the clinical safety of telehealth services using multiple communication tools like chat message and voice calls. Past studies have analyzed telehealth programs without categorizing them by the nature of the program (ie, synchronous and asynchronous) or communication tools [10-15]. However, it is worth noting that studies with a single communication tool reported no clinical differences between traditional and telehealth approaches, which is logically consistent with the findings in our study. Nevertheless, as video calls were associated with only approximately 100 cases and no outcomes, adequate evaluation could not be conducted using regression analysis in this study. We hope to reevaluate the safety of video calls when more data are available.

### Limitations

The generalizability of this study is subject to certain limitations, owing to the characteristics of the study population. The age distribution of the participants was slightly different from that of the nationwide population. According to a recent government report, the age distribution of pregnant women in Japan is as follows: 0.83% for those under 20 years of age, 33.84% for those between 20 and 29 years, 59.44% for those aged 30-39 years, and 5.90% for those aged 40 years or older [20]. Compared to the national distribution, the study participants had a larger proportion of people aged under 20 years and between 20 and 29 years. This may be because using online services are more common among the younger generation. Additionally, the service was funded by the government and became available for those who were not members of client organizations between May and August 2020. This could have influenced the user demographics during the study period because the awareness about the service might have increased among the younger generation, who are less likely to work in a corporation or be involved in pregnancy and childbirth. Thus, the findings are limited to this telehealth service and may not be generalized to other telehealth services.

Other limitations of this study need to be acknowledged, particularly in data collection. First, the survey response rate was 40.9%, which may be insufficient to evaluate the behavioral patterns of all service users. Second, due to the small number of video call consultation cases, the analysis could have insufficient statistical power. Third, users’ selection of communication tools may be subject to self-selection bias. The very low number of users who chose video calls may be explained by users’ circumstances during the call, such as not having sufficient equipment or calling while on the go, when they could not use the camera. Fourth, although the survey queried participants’ emergency visits and hospitalization at night after service use, the outcomes may contain hospital visits that are not technically emergencies, which may have led to overestimation. Fifth, the survey questions were not validated to ensure a precise record of patients’ hospital visits after service use. Sixth, we could not analyze multiple hospital visits after service use because neither the survey items nor other monitoring protocols tracked the study participants’ frequency of and reasons for hospital visits after the consultation. Other variables, such as medical history, pregnancy and delivery situations, gestational weeks of pregnancy, socioeconomic status, and family environment could also not be adjusted for this study due to the lack of data. In addition, the final pregnancy outcomes could not be evaluated in this study. Therefore, further research needs to examine the clinical outcomes of telehealth services more closely and with larger data sets, including these contextual factors and the course of pregnancy. Specifically, the use of validated questionnaires and monitoring protocols to document detailed information regarding patients’ hospital visits after service use is encouraged.

### Conclusions

Our analysis suggests that different communication tools for telehealth services in obstetrics and gynecology may not be associated with clinical safety among service users. However, there were several limitations, and the results require interpretation in light of the characteristics of telehealth service provided to the participants. Future research should analyze the data with more emergency cases and relevant variables to examine the consequences of synchronous telehealth consultation services.

## Abbreviations

OR: odds ratio
